# First Order Temperature Dependent Phase Transition in a Monoclinic Polymorph Crystal of 1,6-Hexanedioic Acid: An Interpretation Based on the Landau Theory Approach

**DOI:** 10.3390/molecules190710137

**Published:** 2014-07-11

**Authors:** Hoong-Kun Fun, Suchada Chantrapromma, Lye-Hock Ong

**Affiliations:** 1 X-ray Crystallography Unit, School of Physics, Universiti Sains Malaysia, 11800 USM, Penang, Malaysia; 2 Department of Pharmaceutical Chemistry, College of Pharmacy, King Saud University, Riyadh 11451, Kingdom of Saudi Arabia; 3 Department of Chemistry, Faculty of Science, Prince of Songkla University, Hat-Yai, Songkhla 90112, Thailand

**Keywords:** adipic acid, crystal structure, Landau theory, phase-transition

## Abstract

Crystals of 1,6-hexanedioic acid (**I**) undergo a temperature-dependent reversible phase transition from monoclinic *P*2_1_/c at a temperature higher than the critical temperature (*T*_c_) 130 K to another monoclinic *P*2_1_/c at temperature lower than *T*_c_. The phase transition is of first order, involving a discontinuity and a tripling of the *b*-axis at *T*_c_ whereas the other unit cell parameters vary continuously. The transition is described by the phenomenological Landau theory. The crystal structure analyses for data collected at 297(2) K and 120.0(1) K show that there is half of a molecule of (**I**) in the asymmetric unit at 297(2) K whereas there are one and a half molecules of (**I**) in the asymmetric unit at 120.0(1) K. At both temperatures, 297(2) and 120.0(1) K, intermolecular O-H···O hydrogen bonds link the molecules of **I** into infinite 1D chains along [101] direction. However there are significantly more O-H···O hydrogen bonds presented in the 120.0(1) K polymorph, thereby indicating this phase transition is negotiated via hydrogen bonds. The relationship of the conformational changes and hydrogen bonding for these two polymorphs are explained in detail.

## 1. Introduction

**Figure 1:**
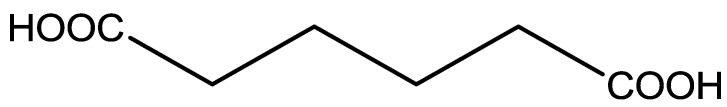
Schematic diagram of adipic acid (**I**).

Reports on several types of phase transitions due to hydrogen bonding [1,2] and their conformation changes [3] have been published. In our previous investigations, we have reported the studies of hydrogen bonding phase transitions in phenol/benzoic acid and amine adducts [4,5,6,7,8]. In those crystals the structural phase transitions have been from monoclinic-to-triclinic [4] and from orthorhombic-to-monoclinic [7]. The phenomenological Landau theory of ferroelastic phase transitions was developed to identify the primary order parameters of these structural phase transitions leading eventually to the clarification of these structural phase transitions [5,6,8]. Owing to our interest in phase transitions of organic compounds both in structural and theoretical studies, we have prepared crystalline forms of 1,6-hexanedioic acid or adipic acid (**I**, Figure 1) which is an interesting aliphatic dicarboxylic acid due to its undulatory behavior in the solid state. A monoclinic polymorph of adipic acid has been reported by Ranganathan, Kulkarni and Rao [9] which did not exhibit polymorphism, but underwent a phase transition. Recently, Ohki, Nakamura and Chihara reported that the adipic acid undergoes a phase transition at about 136 K [10]. However, our study shows that **I** actually exhibits polymorphism in forms of triclinic polymorph [11] and monoclinic polymorph (in this study). Moreover we found that the triclinic polymorph [11] does not undergo phase transition whereas the monoclinic polymorph shows a reversible first-order temperature dependent phase-transition.

In this paper, we report the preparation, temperature-dependent phase transition and the X-ray structural analyses of **I** at 297(2) and 120.0(1) K which will be referred to as room-temperature phase (RTP) and low-temperature phase (LTP), respectively. The phase transition of **I** is described macroscopically by the Landau phenomenological theory approach.

## 2. Results and Discussion

Adipic acid reported in this study crystallizes in a centrosymmetric monoclinic *P*2_1_/c space group. Using the same crystal to collect data at high temperature and low temperature, it was found that the crystal of **I** undergoes a reversible temperature-dependent phase transition from monoclinic *P*2_1_/c at the temperature higher than the critical temperature (*T*_c_) 130 K to another monoclinic *P*2_1_/c at temperature lower than *T*_c_. The crystal data of **I** at 297(2) K (RTP) and 120.0(1) K (LTP) are summarized in Table 1. Selected bond lengths, bond angles and torsion angles of the RTP and LTP structures are listed in Table 2. The interplanar angles between the functional units in **I** are listed in Table 3 and the hydrogen bonds are listed in Table 4.

**Table 1:** Crystal data and parameters for structure refinement of **I** at 297(2) and 120.0(1) K.

Crystal Properties	297(2) K (RTP)	120.0(1) K (LTP)
CCDC deposition numbers	989931	989973
Formula	C_6_H_10_O_4_	C_6_H_10_O_4_
Formula Weight	146.14	146.14
Color; Shape	Colorless; Block	Colorless; Block
Crystal System	Monoclinic	Monoclinic
Space Group	*P*2_1_/c	*P*2_1_/c
Z	2	6
Lattice Constants	*a* =7.3647(3)Å	*a* = 7.3865(6)Å
	*b* = 5.1503(3)Å	*b* = 14.9130(13)Å
	*c* = 10.1332(5)Å	*c* = 10.0475(9)Å
	*β* = 112.274(3)°	*β* = 111.656(6)°
Volume [Å^3^]	355.42(3)	1028.66(15)
D_x_ [Mg·m^−3^]	1.366	1.415
μ [mm^−1^]	0.115	0.120
F(000)	156	468
θ range [°]	2.99–32.50	2.57–27.50
*h*, *k*, *l*	−10/11, −7/7, −14/15	−9/9, −19/7, −13/13
Reflections Collected	5118	10307
Reflections Unique	1279	2346
Tmin/Tmax	0.9661/0.9394	0.9373/0.9649
R(int)	0.0270	0.0523
Number of Parameters	67	136
GoF	1.052	1.180
Final R index[I > 2σ(I)]	0.0453	0.0949

**Table 2:** Selected bond lengths (Å), angles (°) and torsion angles (°) for **I**.

Monoclinic at 297(2) K		Monoclinic at 120.0(1) K	
Bonds			
O1-C1	1.2242(11)	O1A-C1A	1.219(5)
O2-C1	1.2989(11)	O2A-C1A	1.308(5)
O2-H1O	0.98(2)	O2A-H1OA	0.82
C1-C2	1.4931(11)	C1A-C2A	1.523(6)
C2-C3	1.5091(13)	C2A-C3A	1.499(6)
C3-C3 ^i^	1.5135(15)	C3A-C3 ^ii^	1.546(8)
		O1B-C1B	1.220(5)
		O2B-C1B	1.316(5)
		O2B-H1OB	0.82
		C1B-C2B	1.505(5)
		C2B-C3B	1.514(5)
		C3B-C4B	1.514(5)
Angles			
O1-C1-O2	122.79(8)	O1A-C1A-O2A	123.9(4)
O1-C1-C2	122.93(8)	O1A-C1A-C2A	123.5(4)
O2-C1-C2	114.28(8)	O2A-C1A-C2A	112.6(3)
C1-C2-C3	114.80(8)	C1A-C2A-C3A	113.4(3)
		O1B-C1B-O2B	123.1(3)
		O1B-C1B-C2B	123.7(3)
		O2B-C1B-C2B	113.3(3)
		C1B-C2B-C3B	114.7(3)
Torsion angles			
O1-C1-C2-C3	−7.40(15)	O1A-C1A-C2A-C3A	−2.6(6)
O2-C1-C2-C3	172.92(9)	O2A-C1A-C2A-C3A	177.2(3)
C1-C2-C3-C3 ^i^	−174.46(9)	C1A-C2A-C3A-C3A ^ii^	177.6(4)
		O1B-C1B-C2B-C3B	9.6(6)
		O2B-C1B-C2B-C3B	−170.1(3)
		C1B-C2B-C3B-C4B	172.8(3)

symmetry codes: i = −x, −y, −z; ii = −x + 1, −y + 2, −z + 2.

**Table 3:** Interplanar angles between the functional units in **I**.

Phase	Plane	Plane	Interplanar angle (°)
RTP	O1/O2/C1/C2	C2/C3/C2A/C3A	6.95(9)
at 297(2) K			
LTP	O1A/O2A/C1A/C2A	C2A/C3A/C2AA/C3AA	4.2(4)
at 120.0(1) K	O1B/O2B/C1B/C2B	C2B/C3B/C4B/C5B	8.1(4)
	O3B/O4B/C5B/C6B	C2B/C3B/C4B/C5B	9.3(4)

**Table 4:** Geometries of intermolecular hydrogen bonds in **I**.

Crystals	D-H···A	D-H (Å)	H···A (Å)	D···A (Å)	D-H···A (°)
RTP	O2-H1O···O1 ^iii^	0.985(19)	1.673(19)	2.6508(11)	171.3(18)
at 297(2) K					
LTP	O2A-H1OA···O1A ^iv^	0.82	1.85	2.668(4)	176
at 120.0(1) K	O2B-H1OB···O3B ^v^	0.82	1.83	2.648(4)	175
	O4B-H2BA···O1B ^vi^	0.82	1.84	2.658(4)	175

Symmetry codes: iii = 1 − x, −y, 1 − z; iv = −x, 2 − y, 1 − z; v = −1 + x, y, −1 + z; vi = 1 + x, y, 1 + z.

### 2.1. Crystal Structure

**Figure 2:**
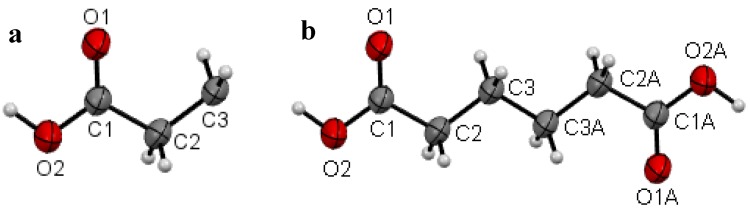
(**a**) Asymmetric unit of **I** with the atomic numbering schemes at 297(2) K (RTP) and (**b**) molecular view of **I** with the atomic numbering schemes at 297(2) K (RTP). The atoms with suffix “A” were generated by symmetry code −x, −y, −z.

**Figure 3:**
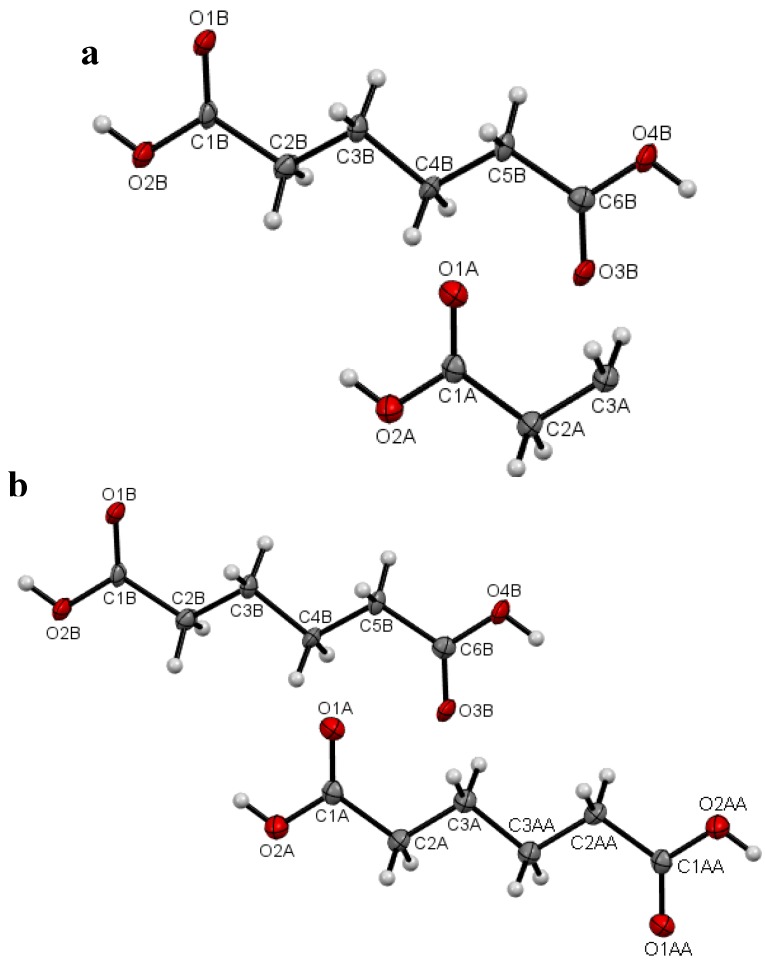
(**a**) Asymmetric unit of **I** with the atomic numbering schemes at 120.0(1) K (LTP) and (**b**) molecular view of **I** with the atomic numbering schemes at 120.0(1) K (LTP). The atoms with suffix “AA” were generated by symmetry code –x + 1, −y + 2, −z + 2.

Plots of an asymmetric unit of **I** at the temperature 297(2) K (RTP) and 120.0(1) K (LTP) are shown in Figure 2a, b and Figure 3a, b respectively. The asymmetric unit of the RTP structure consists of half a molecule (Figure 2a), whereas there are one and a half crystallographic independent molecules in the asymmetric unit of the LTP structure (Figure 3a). In the RTP molecular structure which is shown in Figure 2b, there is an inversion center on the central C-C bond and the molecular backbone adopts the expected planar zig-zag structure. For the LTP molecular structure which is shown in Figure 3a, the half-molecule fragment resembles the asymmetric unit of the RTP. However the LTP molecule is relatively more planar (compared to the RTP molecule) with the torsion angle C1A-C2A-C3A-C3A^ii^ being 177.6(4)° and the angle made by the carboxylic acid O1A-C1A-C2A-C3A = −2.6(6)° (Figure 3b), the corresponding angles being −174.46(9) and −7.40(15)° in the RTP (Table 2). The molecular back bone of the full molecule fragment of the LTP is buckled with the torsion angles C1B-C2B-C3B-C4B = 172.8(3)°, C2B-C3B-C4B-C5B = 176.1(3)° and C3B-C4B-C5B-C6B = −174.0(3)°.

The bond lengths and angles in RTP and LTP structures are in normal ranges and agree with the corresponding values in the triclinic polymorph [11]. The interplanar angles of the functional groups in the temperature polymorphs are listed in Table 3. With temperature changes, the bond lengths and angles of **I** are almost unchanged in the molecule with half symmetry (half-molecule fragment) which can be seen in (Table 2). However even though these parameters are slightly different in the full-molecule fragment (molecule *B* in Figure 3b) which lost its symmetry but the torsion angles are significantly different, resulting in significant conformational changes. As can be seen in Table 4, the RTP structure has one O-H···O hydrogen bond whereas the LTP structure has three O-H···O hydrogen bonds. Their geometries and symmetry operations are listed in Table 4. The extra hydrogen bonds, *i.e.*, O2B-H1OB···O3B^v^ and O4B-H2OB···O1B^vi^ (Table 4) interconnecting the molecule of LTP structure are not observed in the RTP structure.

**Figure 4:**
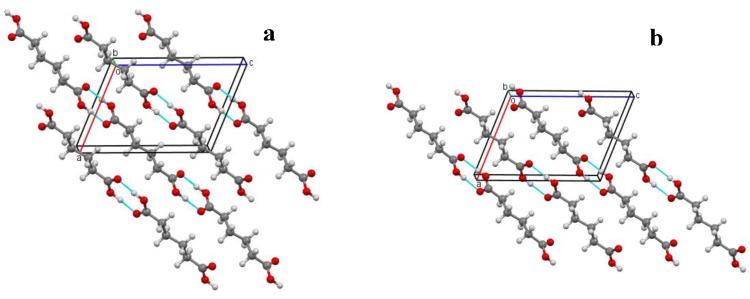
Packing diagram of **I** at (**a**) 297 (2) K (RTP) and (**b**) 120.0 (1) K (LTP) viewed down the *b*-axis. Hydrogen bonds are drawn as dash lines.

In the crystal packing of both RTP and LTP structures, the molecules are linked via centrosymmetric pairs of intermolecular O-H···O hydrogen bonds, forming infinite one-dimensional chains along the [101] direction (Figure 4a,b). These chains are further stacked along the *b*-axis.

Summarizing, these extra O-H···O hydrogen bonds presented in the LTP structure initiates the reversible temperature-dependent phase transformation leading to the conformational changes of the full-molecule fragment structure in the LTP.

**Figure 5:**
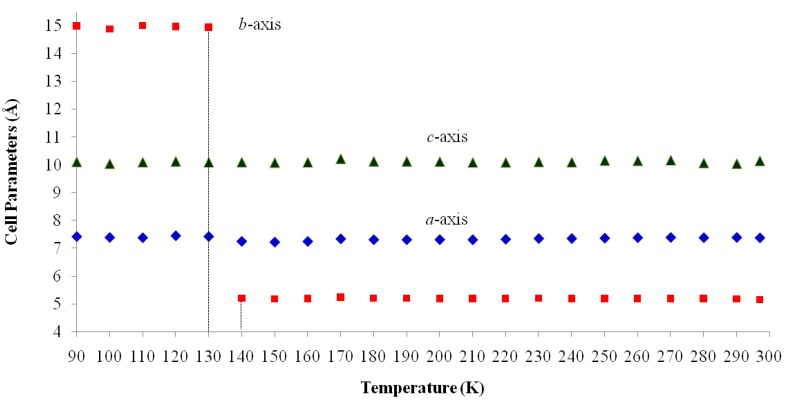
Temperature dependence of cell lengths (*a*, *b* and *c*-axis) of **I**. Cell Parameters (Å) *vs.* Temperature (K).

**Figure 6:**
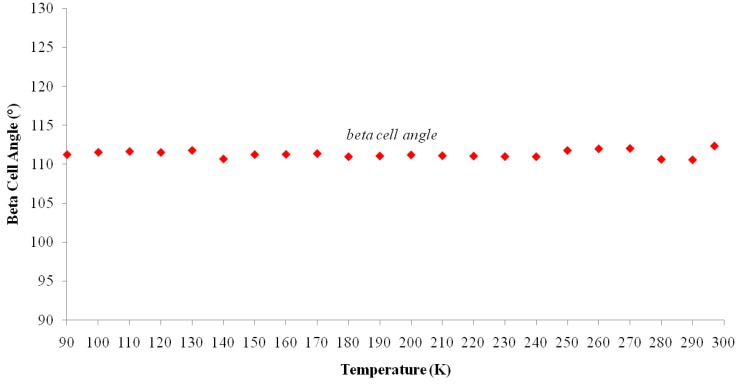
Temperature dependence of angle *β* of **I**. Beta Cell Angle (°) *vs.* Temperature (K).

**Figure 7:**
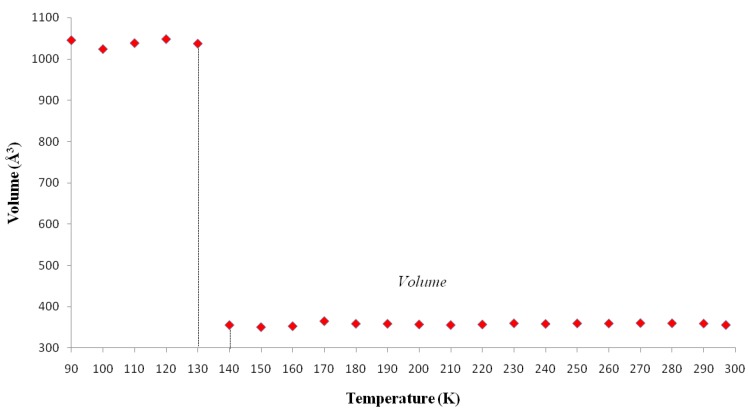
Temperature dependence of volume of **I**. Volume (Å3) *vs*. Temperature (K).

The cell parameters of **I** were measured in the temperature range from 297(2) to 90.0(1) K (Table 5) and the plot is depicted in Figure 5. It is clearly seen that there is a discontinuous jump of the *b* parameter at 130 K. As the temperature is decreased from the room temperature to close to transition temperature *T_c_* = 130 K, the *a* parameter increases by ~0.05 Å while the *b* and *c* parameters show only small variations (Figure 5). Below the transition temperature, the cell parameters is still monoclinic with the same space group and the *a* and *c* parameters remains nearly the same. Surprisingly that the *b* parameter changes to 14.942(11) Å which is almost triple of its value at room temperature (Table 5 and Figure 5). The β angle shows only slight variation on cooling to the low temperature (Figure 6). These data show that the phase transition is of first order, involving a discontinuity and a tripling of the *b*-axis at *T_c_* whereas the *a*-axis and *c*-axis are continuous and essentially unchanged. In addition, the β angle (Figure 6) also varies insignificantly during transition. The temperature dependence of the unit cell volume, which was shown in Figure 7, almost triple on cooling in accordance to the changes of the cell parameters. 

**Table 5:** Temperature dependent cell parameters for **I**.

Temp (K)	*a* (Å)	*b* (Å)	*c* (Å)	*β* (°)	Volume (Å^3^)
297	7.3660(4)	5.1539(3)	10.1383(5)	112.333(4)	356.0(2)
290	7.38(3)	5.182(18)	10.04(4)	110.56(7)	359.5(1)
280	7.37(3)	5.19(2)	10.06(5)	110.62(9)	360.2(1)
270	7.38(2)	5.193(16)	10.16(3)	112.02(9)	360.6(1)
260	7.37(2)	5.191(13)	10.14(3)	111.97(8)	359.6(9)
250	7.355(17)	5.191(11)	10.15(2)	111.76(7)	359.8(9)
240	7.342(15)	5.188(10)	10.09(2)	110.96(5)	358.7(9)
230	7.342(13)	5.198(9)	10.10(2)	110.98(4)	360.0(8)
220	7.315(14)	5.188(9)	10.08(2)	111.04(5)	357.2(8)
210	7.299(13)	5.185(9)	10.08(2)	111.09(4)	355.9(8)
200	7.302(16)	5.194(11)	10.11(2)	111.19(7)	357.3(8)
190	7.30(2)	5.202(14)	10.12(3)	111.05(11)	358.9(7)
180	7.30(2)	5.206(15)	10.12(3)	110.96(10)	359.0(8)
170	7.33(3)	5.24(2)	10.21(5)	111.36(11)	365.3(7)
160	7.239(8)	5.187(5)	10.088(14)	111.26(3)	353.0(6)
150	7.215(7)	5.179(5)	10.074(14)	111.23(3)	350.8(6)
140	7.243(16)	5.206(11)	10.09(2)	110.67(10)	355.8(6)
130	7.414(5)	14.942(11)	10.087(9)	111.77(3)	1037.8(3)
120	7.44(2)	14.97(4)	10.12(3)	111.51(5)	1048.7(7)
110	7.37(4)	15.01(9)	10.09(6)	111.63(15)	1039.0(8)
100	7.3791(3)	14.8734(7)	10.0347(5)	111.525(2)	1024.52(7)
90	7.41(3)	14.99(7)	10.10(5)	111.23(9)	1046.0(7)

### 2.2. Landau Phenomenological Theory of First Order Structural Phase Transitions in ***I***

The crystallographic data show that the crystal **I** undergoes an isosymmetric phase transition based on the geometric anomaly from monoclinic *P*2_1_/c at high temperatures (above 140 K) to another monoclinic *P*2_1_/c at low temperatures (below 130 K) without any significant structural change. In order to discuss this anomaly, *Q* is assumed as the order parameter in the Landau phenomenological theory of the structural phase transition in the crystal **I** [12]. At the microscopic level this order parameter may represent a set of displacements, such as conformational changes (which is very obvious from the contents of the asymmetric units in Figure 2b, leading to a tripling of the *b*-axis and a discontinuity in unit cell volume (see Table 2). Thus this isosymmetric phase transition is not brought about by qualitative change in crystal symmetry, but by quantitative change of formation of hydrogen bonds leading to conformational changes. Similar type of phase transition can be induced by quantitative changes such as atomic size, amount of ionic displacement or statistical weight of atomic distribution [13]; and a representative example of isosymmetric phase transition is the γ-α transition in cerium and its alloys [14].

In the present paper on crystal **I**, in order to relate the geometrical quantity of elastic strain with the thermodynamic order parameter *Q* in Landau phenomenological theory, the Landau potential for the crystal **I** is formulated with the bilinear coupling between order parameter *Q* and the elastic strain components. This is assumed that the crystal is in thermodynamic equilibrium with respect to spontaneous strain [12]. In the geometrical representation of monoclinic crystal, the Cartesian coordinate system of axes **X**, **Y**, and **Z** are such that **X** and **Y** are parallel to *a***-**axis and *b***-**axis respectively and **Z** is perpendicular to both these axes and thus it is parallel to c* axis (of the reciprocal lattice). For the notation of elastic strain components, we follow the standard Voigt notation adopted in the theory of elastic deformations of a continuous homogeneous medium [15] to describe the dimensionless strain tensors *e_k_*, with the abbreviated subscript *k* taking integral values from 1 to 6.

The Landau potential *F* is expressed in the power series of order parameter Q and the bilinear coupling with elastic strain components as follow:

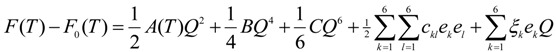
(1)


The term *F*_0_(*T*) is the free energy of the high temperature phase and we adopt the temperature dependence of *A*(*T*) as:

*A*(*T*) = *a*_0_ (*T* − *T*_0_) (2)

where *a*_0_ is a constant and *T*_0_ is the critical temperature. The parameters *B* and *C* are assumed to be weakly temperature dependent over the range of temperatures considered and are therefore taken as constants. The first three terms in (1) represent the Landau potential of the order parameter *Q*. The fourth term represents the quadratic elastic energy per unit volume of a monoclinic crystal, where *c_kl_* is the elastic constants in Voigt notation. The last term is the coupling energy between the order parameter and the elastic strain, with the coupling constants *ξ_k_*. Minimizing Equation (1) with respect to *e_k_*, the simplification leads to 
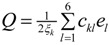
. We can then rewrite *e_k_* in term of *Q* as:

*e_k_* = *λ_k_* (*c_kl_*, *ξ_k_*)*Q* (3)

where *λ*_k_ are the coefficients depending on the elastic constants *ξ_k_* of monoclinic crystal and coupling constants . Eliminating the terms in strain components in Equation (1) using *e_k_* in Equation (3), we obtain the normalized free energy as:

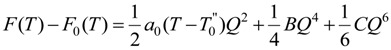
(4)
where 
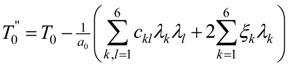
.

Equation (4) is the functional for the usual Landau form of free energy in the first order phase transition if *B* < 0 and *C* > 0 and in this case it is proposed for the phase transition of crystal **I**. The analytical expression for *Q* in terms of the Landau parameters can be obtained by minimizing Equation (4) with respect to *Q* as shown:

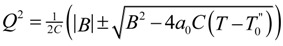
(5)


We have shown in our previous work [8] that the values of *Q*, which correspond to the minima of free energy (given by Equation (5) for *T* < 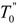), are the values of the spontaneous order parameter (denoted as *Q*'). *Q*' varies non-linearly with temperature *T* with a discontinuity at 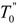 is an indication of first order phase transition.

Table 2 shows the measured lattice parameters for crystal **I** equilibrated at the temperatures from 90 K to 297 K. The temperature variation for the lattice parameters *a*, *b*, *c* and *β* are plotted in Figure 5 to Figure 7. From Figure 5, the abrupt jump in the *b*-unit cell length at the transition temperature may be over-exaggerated owing to the tripling of the unit cell length, however, based on the crystallographic data for temperatures below 130 K, we can estimate the magnitude of this linear strain along the *b*-axis, *e*_2_ as:

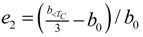
(6)
where *b_<T_C__* is the LTP *b*-unit cell length. The expressions for the other elastic strain components for monoclinic *P*2_1_/c to *P*2_1_/c phase transition in terms of lattice parameters such as 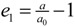, 
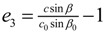
, *e*_4_ = 0, 
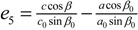
 and *e*_6_ = 0 _,_ are formulated according to Schlenker *et al.* [16]. The values for *a*_0_, *b*_0_, *c*_0_ are the average values of the HTP *a*-unit cell, *b*-unit cell and c-unit cell length respectively. *β*_0_ is the average value of HTP, and as variation of *β* from LTP to HTP is very small, thus it is considered in the calculation that *β* ≈ *β*_0_. The scalar spontaneous strain *e_s_*, according to that proposed by Aizu [17] is:

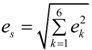
(7)


With respect to the structural properties of monoclinic symmetry in crystal **I**, the spontaneous strain from Equation (6) will be reduced to 
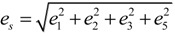
. Since from the data *β* ≈ *β*_0_, *e*_3_ and *e*_5_ ≈ 0, thus 
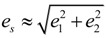
.

**Figure 8:**
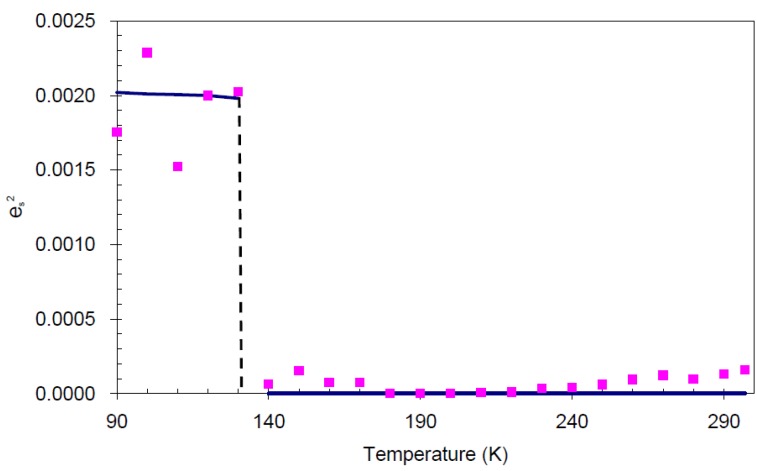
Temperature variation of 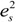 (square of spontaneous strain). The line indicates the trend of the data, and the values are calculated from raw data.

In order to establish the linear relationship between the strain components and *Q*, which gives strong indication that crystal **I** undergoes first order isosymmetric phase transition, a plot of the square of spontaneous strain *e^2^* versus temperature is shown in Figure 8. The graph shows a clear discontinuity at the transition temperature (130 K) that corresponds to the discontinuity in the *b*-unit cell length (Figure 5) and unit cell volume shown (Figure 7). Since the elastic strain components are linearly proportional to *Q* as shown in Equation (3), thus the discontinuity of the geometric quantities in crystal **I** implies the first order isosymmetric phase transition of crystal **I**. Although the geometric anomalies of tripling of *b*-unit cell length and unit cell volume may be an exaggerated picture of a first order phase transition in crystal **I**, however, the above analysis is an indirect prediction based on the discontinuities of 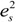 and order parameter *Q^2^* [12].

## 3. Experimental Section

### General Information

Adipic acid or 1,6-hexanedioic acid (**I**) was obtained commercially (Fluka, Buchs, Switzerland). Crystals of 1,6-hexanedioic acid were grown by the slow evaporation from ethyl acetate solution to afford the colorless block-shaped single crystals of **I**. The crystal obtained was subjected on X-ray structural analyses. The single crystal of 0.30 × 0.52 × 0.55 mm^3^ in size was mounted on a glass fiber with epoxy cement for X-ray crystallographic study. Using the same crystal, the cell parameters were measured in the temperature range from 297(2) to 90.0(1) K. Two crystallographic data of **I** were collected at 297(2) and 120.0(1) K, respectively, and these crystallographic data and experimental details are presented in Table 1. The data were collected using a Bruker APEX2 CCD diffractometer with a graphite monochromated MoK_α_ radiation at a detector distance of 5 cm and with *APEX2* software [18]. Crystallographic data at 120.0(1) K were collected with the Oxford Cyrosystem Cobra low-temperature attachment. The collected data were reduced using *SAINT* program [18] and the empirical absorption corrections were performed using the *SADABS* program [18]. The structures were solved by direct methods and refined by least-squares using the *SHELXTL* software package [19]. For the RTP polymorph, all non-hydrogen atoms were refined anisotropically and all H atoms were located from difference Fourier maps and isotropically refined. For the LTP polymorph, all H atoms were placed in calculated positions with distances of O–H = 0.82 Å and C–H = 0.97 Å after checking their positions in the difference map. The *U_iso_* values were constrained to be 1.2*U_eq_* of the carrier atoms. The final refinement converged well. The selected bond lengths are presented in Table 2. Materials for publication were prepared using *SHELXTL* [19], *PLATON* [20] and Mercury [21]. The crystallographic-information files for **I** at temperature 297(2) and 120.0(1) K have been deposited in the Cambridge Crystallographic Data Base Center with deposition numbers CCDC989931 and CCDC989973, respectively. 

## 4. Conclusions

The cell parameters of crystal **I** were measured in the temperature range from 297 to 90 K. The transition occurs at *T_C_* = 130 K. These data show that the isosymmetric phase transition is of first order, involving discontinuities and a tripling of the *b*-axis and cell volume at *T_C_* (Figure 4a). The analysis of bilinear coupling between order parameter Q and elastic strain components formulated in the Landau potential shows that the Landau phenomenological theory of first order structural phase transition in crystal **I** is adequate in illustrating the feature of phase transition through the spontaneous strain. These spontaneous strain is caused by the conformational changes of the molecular structure resulting from extra hydrogen bonding at the low temperature structure which has initiated the first order reversible temperature-dependent phase transition in crystal **I**.
